# Impact of sex on the outcome of troponin-positive patients with non-obstructive coronary arteries

**DOI:** 10.1038/s41598-025-10932-z

**Published:** 2025-07-22

**Authors:** Fabienne Kreimer, Clara Schlettert, Mohammad Abumayyaleh, Ibrahim Akin, Mido Max Hijazi, Michael Gotzmann, Nazha Hamdani, Andreas Mügge, Assem Aweimer, Ibrahim El-Battrawy

**Affiliations:** 1 Department of Cardiology and Rhythmology, University Hospital St. Josef Hospital Bochum, Ruhr University Bochum, Bochum, Germany; 2 Department of Cardiology and Angiology, Bergmannsheil University Hospital, Ruhr University of Bochum, Bürkle de la Camp-Platz 1, 44789 Bochum, Germany; 3 First Department of Medicine, University Medical Centre Mannheim (UMM), Mannheim, Germany; 4 Division of Spine Surgery, Department of Neurosurgery, Technische Universität Dresden, Faculty of Medicine, and University Hospital Carl Gustav Carus, Dresden, Germany; 5 Department of Cellular and Translational Physiology, Institute of Physiology, Institut für Forschung und Lehre (IFL), Molecular and Experimental Cardiology, Ruhr-University Bochum, Bochum, Germany; 6 Department of Cardiology II - Rhythmology, University Hospital Muenster, Muenster, Germany

**Keywords:** Risk factors, Cardiology, Interventional cardiology

## Abstract

The aim of this study was to investigate the prognostic impact of sex on in- and out-of-hospital adverse events in troponin-positive patients with non-obstructive coronary artery disease (CAD). 24,775 patients who underwent coronary angiography from 2010 to 2021 were screened for this study. The final study population consisted of 373 troponin-positive patients with non-obstructive CAD with a follow-up period of 6.2 ± 3.1 years, with 185 males and 188 females. The primary study end point was a composite of in-hospital adverse events. Secondary endpoints covered out-of-hospital adverse events during follow-up. In-hospital adverse event rates revealed no significant sex differences (37.8% in males vs. 33.0% in females). Significantly more long-term adverse events occurred in women compared with men during follow-up (27.3% vs. 41.9%). All-cause mortality was significantly higher in women than in men (29.7% vs. 21.2%, *p* = 0.022). Cox analysis identified age ≥ 70 years, arterial hypertension, diabetes mellitus, supraventricular tachycardia, pulmonary disease, neurological disease, and kidney disease as predictors of out-of-hospital adverse events, whereas male sex was associated with a better long-term outcome. While sex differences were not significant in in-hospital adverse events, females demonstrated a higher incidence of out-of-hospital adverse events and increased mortality during long-term follow-up compared to males.

## Introduction

Myocardial infarction with non-obstructive coronary arteries (MINOCA) is a clinical scenario characterized by a patient displaying symptoms indicative of acute coronary syndrome, elevated troponin levels, and non-obstructive coronary arteries during coronary angiography (defined as coronary artery stenosis < 50% in any major epicardial vessel)^1^. The prevalence of MINOCA varies widely in different studies, ranging from approximately 1–14% of patients with acute coronary syndrome undergoing coronary angiography^1,2^.

MINOCA is a comprehensive term encompassing a diverse array of underlying conditions, resulting in a highly heterogeneous patient population^2^. Conditions include both coronary and non-coronary pathologies, involving cardiac as well as extracardiac disorders^1,2^. Following coronary angiography without evidence of coronary artery stenosis, MINOCA is considered a working diagnosis rather than a definitive diagnosis^1,2^. Clinicians are urged to conduct additional evaluations and investigations, including further imaging studies like magnetic resonance imaging (MRI), to uncover the cause of MINOCA and establish a more definitive diagnosis, leading to appropriate patient treatment^1,2^.

Research indicates that MINOCA patients, in comparison to those with myocardial infarction and obstructive coronary artery disease (CAD), are typically younger, more frequently non-obese, non-smokers, not have arterial hypertension or chronic kidney disease, in summary, lack traditional cardiovascular risk factors^3–10^. The prognosis for MINOCA patients appears more favourable, with a one-year mortality rate of 3.5%, in contrast to myocardial infarction patients with CAD, who face a one-year mortality rate of 6.7%^7,11^. However, despite this, the survival rate of MINOCA patients remains inferior when compared to that of healthy individuals^7^.

An analysis of the VIRGO study aimed to compare clinical characteristics and outcomes among young patients with MINOCA versus those with myocardial infarction and obstructive CAD^10^. Among 2690 patients who underwent coronary angiography, 88.4% had myocardial infarction with obstructive CAD, 11.1% had MINOCA, and 0.6% could not be classified^10^. Women had a significantly higher likelihood of experiencing MINOCA compared to men, with a prevalence of 14.9% versus 3.5%^10^.

However, the impact of sexes on the short-term and long-term outcome in patients with MINOCA is unknown.

Therefore, the aim of the present study was to investigate the prognostic impact of sex on in- and out-of-hospital-hospital adverse events and long-term outcomes, including mortality, in troponin-positive patients with non-obstructive coronary arteries.

## Methods

In this study, all patients who underwent coronary angiography at Bergmannsheil University Hospital between January 2010 and April 2021 were examined. Patients gave informed consent. All patients had a medical history, medication, laboratory results, ECG, and echocardiography during hospitalisation.

This study was a monocentric retrospective analysis of clinical derived data. The study was approved by the local ethics committee of the Ruhr University Bochum and was performed in accordance with the Declaration of Helsinki.

### Inclusion and exclusion criteria, follow-up, and study endpoints

All troponin-positive patients who met the criteria for non-obstructive CAD after coronary angiography were included in this study. MINOCA served as a working diagnosis in these patients: First, cardiac troponin levels had to be increased or decreasing, with at least one value above the 99th percentile. Second, clinical evidence of myocardial infarction had to be present, as evidenced by at least one of the following conditions: Symptoms of myocardial ischemia, new ischemic changes on electrocardiogram, pathological Q waves, evidence of new loss of viable myocardium or new regional wall motion, abnormalities suggestive of an ischemic cause, or evidence of coronary thrombus by angiography or autopsy. In addition, patients undergoing coronary angiography required exclusion of coronary artery obstruction (stenosis < 50%)^1,2^.

The diagnosis of troponin-positive with non-obstructive CAD was made independently by an experienced cardiologist and a graduate student based on evaluation of coronary angiograms, echocardiograms, ECGs, and laboratory reports.

The final included patients were then divided into a male and female cohort for analysis.

Patients were excluded if alternative diagnoses were conceivable that could have caused the clinical presentation of troponin elevation. In addition, patients with pre-existing obstructive CAD, patients younger than 18 years and those with incomplete data sets were excluded from the study. Another exclusion criterion was the presence of severe concomitant diseases that significantly limited life expectancy (< 2 years), such as advanced tumour disease.

The primary study end point was the occurrence of in-hospital adverse events, which included a combination of stroke, cardiopulmonary resuscitation, cardiogenic shock, pulmonary oedema, invasive and non-invasive ventilation, left ventricular thrombus, thromboembolic events, life-threatening arrhythmias, supraventricular arrhythmias, and all-cause mortality.

The secondary end point was the occurrence of out-of-hospital adverse events (during follow-up), a combination of stroke, thromboembolic events, recurrence of troponin-positive with non-obstructive CAD, percutaneous coronary intervention, cardiac arrest, and all-cause mortality.

A follow-up was performed between May and September 2023. For this purpose, the data following presentations/hospitalisations in the university clinic and/or a telephone contact with the patients were used. In the case of deceased patients, contact was conducted with the patients’ primary care physicians.

### Statistics

Statistical analysis was performed using SPSS Statistics 23.0 software. Continuous variables with normal distribution were presented as mean ± standard deviation, whereas continuous variables with non-normal distribution were presented as median (interquartile range). Categorical variables were reported as number as well as relative frequency (%). The Kolmogorov-Smirnov test was used to assess normality. Continuous variables with normal and nonnormal distributions were compared using Student’s t test for independent samples or Mann-Whitney U test. Categorical variables were compared with either the chi-square test or Fisher’s exact test. Cox regression analysis was used to identify independent predictors of out-of-hospital adverse events for male and female. Kaplan-Meier analyses were used to assess the prognostic impact of sex on the outcome. A p value < 0.05 was considered significant. All probability values reported are 2-sided.

## Results

### Baseline characteristics of the cohort

**Fig. 1:**
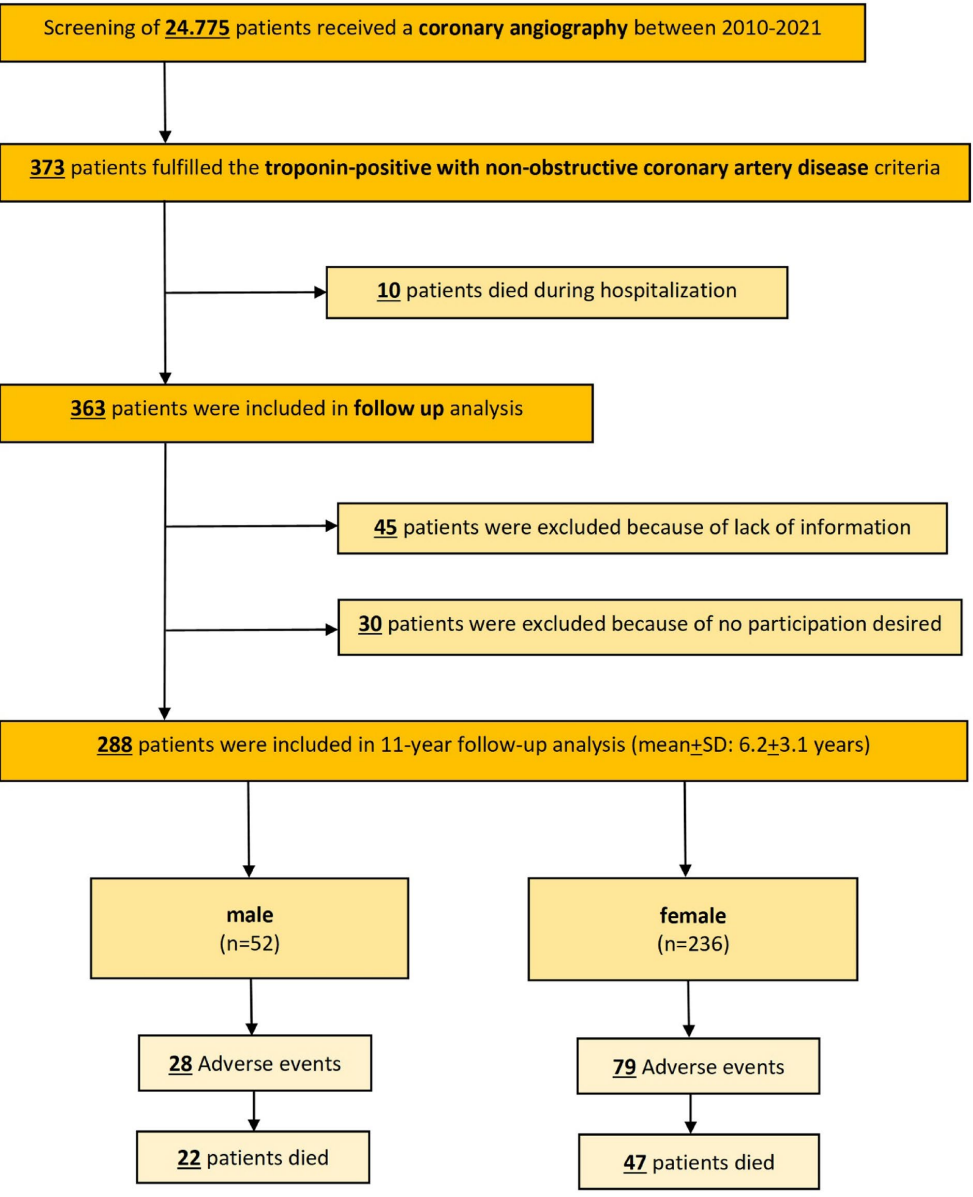
Flow chart presenting the screened data and included patients.

24,775 patients who underwent coronary angiography from 2010 to 2021 were screened for this study (Fig. 1). The final study population consisted of 373 troponin-positive patients with non-obstructive CAD with a follow-up period of 6.2 ± 3.1 years, including 185 males and 188 females. Notably, there was a significant age difference between the two groups, with males averaging 59 years (± 17 years) and females 67 years (± 12 years).

There was a slightly higher incidence of angina pectoris in females (66%) compared to males (57.1%), although this difference did not reach statistical significance. The presence of dyspnoea and palpitations showed no substantial gender-based variation.

No significant gender-based differences were observed in systolic and diastolic blood pressure or heart rate.

The ECG data revealed a notable disparity in the prevalence of ST-segment elevation, with 21.1% in males compared to 8.5% in females.

A gender-based contrast emerged in the medical history regarding smoking, with 30.6% of males having a smoking history compared to 15.7% of females. Additionally, a history of malignancy exhibited a statistically significant difference.

Creatine phosphokinase, troponin, BNP, Creatinine, thyroid-stimulating hormone, free triiodothyronine, and free thyroxine levels were similar between the sexes.

Echocardiographic parameters, including left ventricular ejection fraction (LVEF), the presence of left ventricular hypertrophy and cardiac valve abnormalities, did not show significant gender-based differences. The mean LVEF in males was 38.2% and in females 35.6% which increased in both groups to 49.9% and 48.1% at follow-up (Table 1).

**Table 1:** Baseline characteristics of 373 troponin-positive patients with non-obstructive coronary artery disease.

Variables	Male (*n* = 185)	Female (*n* = 188)	*p* value
Demographics			
Age, mean ± SD	59 ± 17	67 ± 12	**< 0.001**
Symptoms, n (%)			
Angina pectoris	104 (57.1)	122 (66)	0.083
Dyspnoa	80 (43.7)	84 (45.2)	0.780
Palpations	27 (14.8)	18 (9.7)	0.136
Clinical parameters			
Systolic BP, mmHg	144 ± 29	149 ± 87	0.345
Diastolic BP, mmHg	85 ± 20	84 ± 16	0.606
Heart rate, bpm	92 ± 30	88 ± 27	0.186
ECG data, n (%)			
ST-segment elevation	39 (21.1)	16 (8.5)	**< 0.001**
Inversed T-Waves	86 (46.5)	97 (51.9)	0.299
Medical history, n (%)			
Smoking	56 (30.6)	29 (15.7)	**< 0.001**
Diabetes mellitus	34 (18.5)	31 (16.5)	0.614
Obesity (BMI > 30 kg/m^2^)	59 (31.9)	53 (28.2)	0.436
Hypertension	120 (65.2)	133 (70.7)	0.222
COPD	22 (12)	25 (13.3)	0.697
Bronchiale asthma	16 (8.7)	17 (9)	0.906
History of malignancy	17 (9.2)	30 (16.1)	**0.047**
Neurological disease	37 (20.1)	53 (28.3)	0.064
Kidney disease	24 (13.1)	29 (15.4)	0.511
Autoimmune disease	9 (4.9)	8 (4.3)	0.778
Atrial fibrillation	24 (13)	33 (17.7)	0.219
Atrial flutter	0 (0)	0 (0)	0
Laboratory values, median ± IQR			
Troponin (µg/L)	0.1 ± 1.3	0.4 ± 1.4	0.078
Creatine phosphatkinase (µmol/sL)	3.9 ± 3	2.3 ± 2.9	0.055
BNP (pmol/L)	11.6 ± 14.7	29.2 ± 84.8	0.187
Creatinine ( µmol/L )	88 ± 30.8	79.2 ± 41.8	0.232
TSH (mIU/L)	1.2 ± 1.4	1 ± 2.5	0.149
fT3 (pmol/L)	5.7 ± 0.9	5.1 ± 1.6	0.581
fT4 (pmol/L)	15.4 ± 5.1	13.5 ± 4.2	0.803
Echocardiography data, n (%)			
Left ventricular EF % (LVEF)	38.2 ± 24	35.6 ± 26.9	0.335
Follow-up EF% (LVEF)	49.9 + 14.8	48.1 + 15	0.764
Left ventricular hypertrophy (LVH)	54 (30.3)	49 (27.8)	0.605
Drugs on admission, n (%)			
Beta-blocker	56 (30.4)	75 (39.9)	0.056
ACE inhibitor	65 (35.5)	56 (29.8)	0.239
Angiotensin receptor inhibitor	20 (10.9)	37 (19.7)	**0.018**
Calcium channel blocker	37 (20.1)	37 (19.7)	0.918
Diuretics	45 (24.5)	56 (29.8)	0.248
A2-Agonist	4 (2.2)	10 (5.3)	0.172
Anticoagulants*	23 (12.5)	35 (18.6)	0.104
Aspirin	37 (20.1)	42 (22.3)	0.599
Antiarrhythmics**	6 (3.3)	4 (2.1)	0.539

ACE, Angiotensin converting enzyme; BMI, body-mass-index; BNP, brain natriuretic Peptid; BP, blood pressure; COPD, Chronic obstructive pulmonary disease; ECG, Electrocardiogram; LVEF, left ventricular ejection fraction; SD, Standard deviation; *, cumarine, heparin, selective factor 10-blocker, direct thrombin inhibitors; **, Ivabradin, Flecainid, Sotalol, Dronedaron, Digitalis

Significant values are in [bold].

### Medication on admission and at discharge

The percentage of male and female patients receiving beta-blockers increased from 30.4% to 39.9% at admission to 74.1% and 70.7% at discharge, with no statistically significant difference between the sexes.

Similarly, the percentage of patients prescribed with angiotensin converting enzyme inhibitors increased from 35.5% (male) and 29.8% (female) at admission to 64.9% (male) and 55.3% (female) at discharge, however with no statistical significance.

In both men and women, there was a slight increase in the prescription of angiotensin receptor inhibitors from admission to discharge (men: from 10.9 to 13%; women: from 19.7 to 20.2%). There was a significant difference between genders at admission.

No significant gender-based differences in the prescription of Ca-blockers, diuretics, anticoagulants, aspirin, clopidogrel, prasugrel, and antiarrhythmics from admission to discharge were observed (Tables 1 and 2).

**Table 2:** Medication at discharge.

Variables	All patients (*n* = 373)	Male (*n* = 185)	Female (*n* = 188)	p value *
β-blocker	270 (72.4)	137 (74.1)	133 (70.7)	0.475
ACE inhibitor	224 (60.1)	120 (64.9)	104 (55.3)	0.060
Angiotensin receptor inhibitor	62 (16.6)	24 (13)	38 (20.2)	0.060
Ca Blocker	100 (26.8)	51 (27.6)	49 (26.1)	0.743
Diuretics	166 (44.5)	82 (44.3)	84 (44.7)	0.945
Anticoagulants**	104 (27.9)	54 (29.2)	50 (26.6)	0.577
Aspirin	175 (46.9)	80 (43.2)	95 (50.5)	0.158
Clopidogrel	68 (18.2)	31 (16.8)	37 (19.7)	0.465
Prasugrel	0 (0)	0 (0)	0 (0)	0
Antiarrhythmics***	26 (7)	14 (7.6)	12 (6.4)	0.653

*p values for the comparison between male and female group; ACE, Angiotensin-converting-enzyme; ** Cumarine, Heparin, selective factor 10-blocker, direct thrombin inhibitors; *** Ivabradin, Flecainid, Sotalol, Dronedaron, Digitalis

### In-hospital adverse events and treatment approaches

In this study, the primary study endpoint was the occurrence of in-hospital adverse events in male and female patients. The overall incidence of in-hospital adverse events revealed no significant difference between the sexes.

**Fig. 2:**
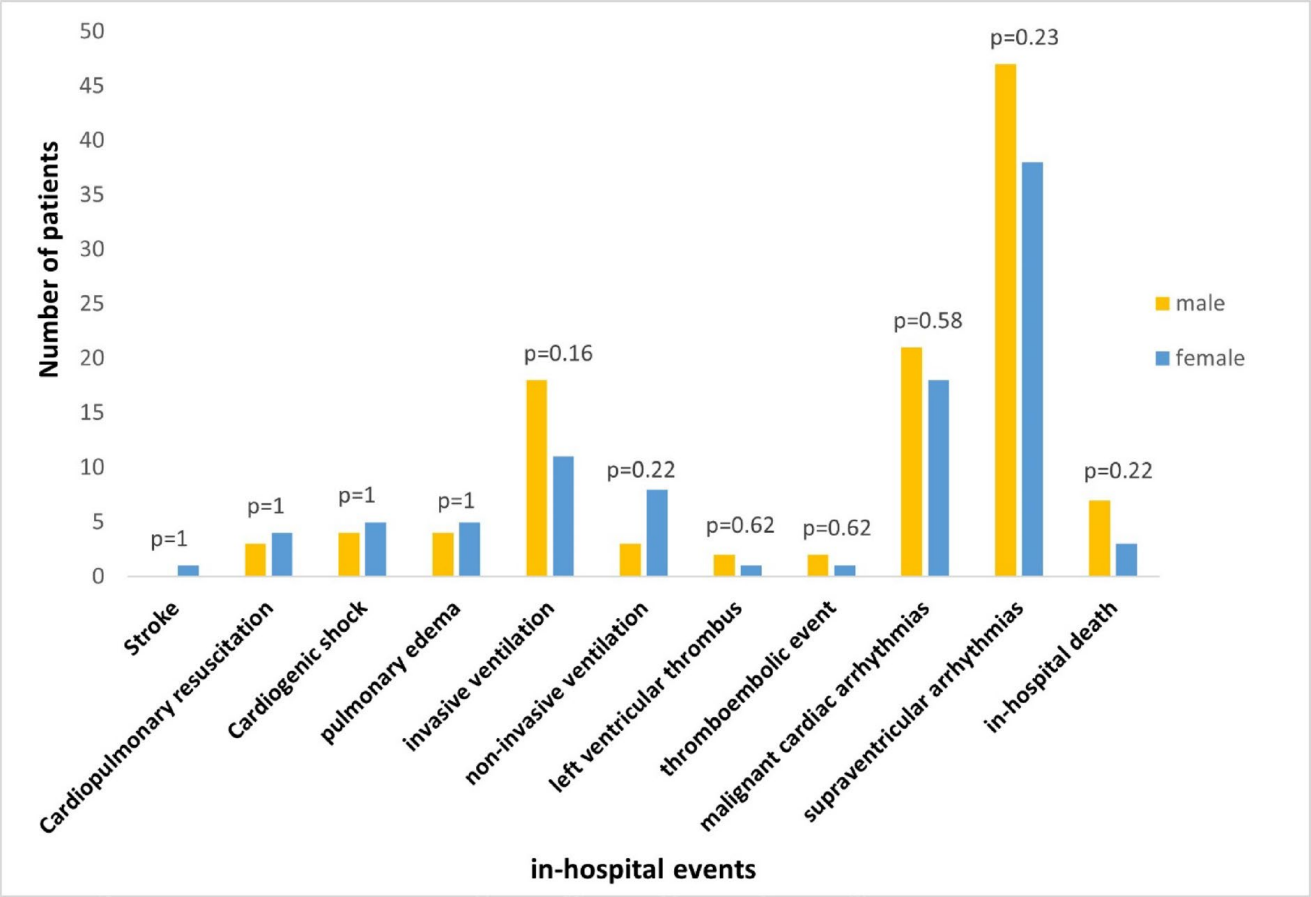
In-hospital adverse events.

Of note, men experienced atrial fibrillation significantly more often (*p* = 0.002) than females. Concerning in-hospital death, the overall incidence was 3.8% in men and 1.6% in women (*p* = 0.217). Further analysis of causes of in-hospital death, cardiac or non-cardiac, revealed no significant gender differences (Fig. 2).

### Out-of-hospital adverse events during follow-up

**Fig. 3:**
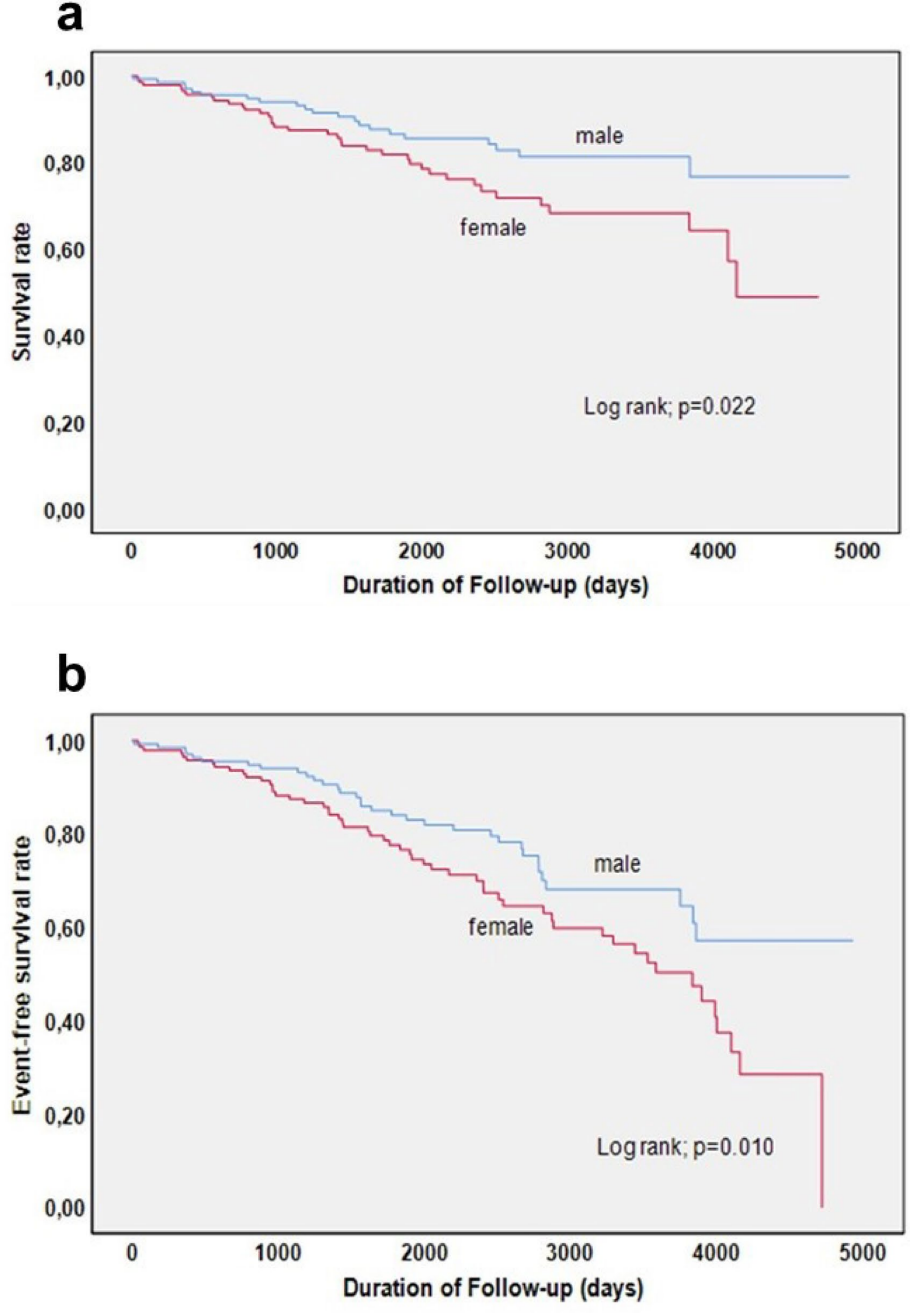
Kaplan-Meier curves representing mortality rates (**a**) and survival free from adverse events (**b**) during follow-up.

The overall incidence of out-of-hospital adverse events during follow-up was significantly higher in females (41.9%) compared to males (27.3%) (*p* = 0.010). While specific events like stroke, thromboembolic events, and recurrence of troponin-positive with non-obstructive CAD demonstrated no significant gender-based differences, overall death and non-cardiac death were significantly higher in females compared to males (overall death: 21.2% of men and 29.7% of women; non-cardiac death: 1.7% of men and 8.6% of women) (Table 3; Fig. 3).

**Table 3:** Out-of-hospital adverse events (during follow up).

Variables	All patients (*n* = 288)	Male (*n* = 140)	Female (*n* = 148)	*p* value *
Adverse event	100 (34.8)	38 (27.3)	62 (41.9)	**0.010**
Stroke	10 (4.2)	6 (5)	4 (3.4)	0.749
Thromboembolic events	6 (2.5)	3 (2.5)	3 (2.5)	1.000
Recurrence of troponin-positive with non-obstructive CAD	3 (1.3)	1 (0.8)	2 (1.7)	0.622
Cardiac arrest	4 (1.7)	3 (2.5)	1 (0.9)	0.622
Percutaneous coronary intervention	17 (7.2)	5 (4.2)	12 (10.3)	0.082
Death	69 (24.1)	25 (21.2)	44 (29.7)	**0.022**
Cardiac caused death	5 (2.1)	3 (2.5)	2 (1.7)	1.000
Non-cardiac caused death	12 (5.1)	2 (1.7)	10 (8.6)	**0.019**

*p values for the comparison between male and female group; adverse event, major adverse cardiac and cerebrovascular events; CAD, coronary artery disease; NSTEMI, Non-ST-segment elevation myocardial infarction; STEMI, ST-segment elevation myocardial infarction; CPR, cardiopulmonary resuscitation

Significant values are in [bold].

Univariate Cox analysis identified age ≥ 70 years (Hazard Ratio (HR) 6.94, 95% Confidence interval (CI) 4.315–11.149, *p* < 0.001), arterial hypertension (HR 2.08, 95% CI 1.259–3.445, *p* = 0.004), diabetes mellitus (HR 2.31, 95% CI 1.437–3.713, *p* < 0.001), supraventricular tachycardia (HR 2.31, 95% CI 1.365–3.901, *P* = 0.002), pulmonary disease (HR 1.92, 95% CI 1.208–3.040, *p* = 0.006), neurological disease (HR 3.02, 95% CI 1.966–4.639, *p* < 0.001) and kidney disease (HR 2.26, 95% CI 1.393–3.679, *p* < 0.001) as predictors of out-of-hospital adverse events. Male sex was associated with a better long-term outcome (HR 0.58, 95% CI 0.379–0.902, *p* = 0.015) (Table 4).

**Table 4:** Cox regression analysis for out-of-hospital adverse events.

Variables	Univariate analysis		
	HR	95% CI	*p* value
Age ≥ 70	6.94	4.315–11.149	**< 0.001**
Male	0.58	0.379–0.902	**0.015**
Arterial hypertension	2.08	1.259–3.445	**0.004**
Diabetes Mellitus	2.31	1.437–3.713	**< 0.001**
BMI > 30 kg/m^2^	1.26	0.809–1.952	0.309
Supraventricular tachycardia	2.31	1.365–3.901	**0.002**
Pulmonary disease	1.92	1.208–3.040	**0.006**
Malignancy	1.43	0.803–2.551	0.224
Neurological disease	3.02	1.966–4.639	**< 0.001**
Kidney disease	2.26	1.393–3.679	**< 0.001**
LVEF < 40%	1.39	0.777–2.491	0.267

BMI, body-mass-index; CI; confidence interval; HR, hazard ratio; LVEF, left ventricular ejection fraction

Significant values are in [bold].

## Discussion

The present study investigated the prognostic impact of sex on in- and out-of-hospital adverse events in 373 troponin-positive patients with non-obstructive CAD. The main findings of our study were:

1. The study detected no significant gender differences in overall in-hospital adverse event rates. However, males had a higher in-hospital rate of atrial fibrillation compared to females.
2. Over follow-up, females experienced a significantly higher incidence of adverse events compared to males.

Several gender-specific differences in cardiovascular disease outcomes were reported^12^. Earlier research indicates that MINOCA patients exhibit clinical traits diverging from the typical profile of individuals susceptible to myocardial infarction with CAD^3^. This group tends to be younger, non-obese, non-smokers, non-diabetic and less likely to suffer from kidney and cerebrovascular disease^3–10^. Furthermore, female gender is highly associated with the occurrence of MINOCA^3,13^. By contrast, in our study, the number of men and women was nearly identical. Evidence is limited regarding an impact of gender on long-term outcome after MINOCA. Indeed, the few studies that have been performed to date on sex differences in MINOCA patients have generated inconsistent results. Recently, several studies demonstrated that the risk for adverse events at follow-up was not sex dependent in MINOCA patients^13–20^. In fact, an analysis of the Swedish Coronary Angiography and Angioplasty Registry observed that women with myocardial infarction and non-obstructive CAD had a lower mortality risk than men^21^. Studies that focused on cardiac imaging also did not demonstrate a gender difference in the outcome of MINOCA patients. Accordingly, in a study involving 1948 patients undergoing combined coronary CT angiography and PET myocardial perfusion imaging, it was demonstrated that there was no interaction between sex and the presence of coronary calcification, non-obstructive CAD, or abnormal stress myocardial blood flow in the prediction of adverse events at follow-up^22^. Moreover, studies even report that the risk of adverse events in MINOCA patients is not only independent of gender, but also low^13,15^.

However, our study obtained different results. First, we detected a total in-hospital adverse event rate of 35.5% and out-of-hospital adverse event rate of 34.8% in the cohort, implying that more than one in three suffered an adverse event during both in-hospital stay and follow-up. Second, we noted a significant sex difference in adverse event rates: while in-hospital outcome was comparable between men and women, women had significantly higher rates of out-of-hospital adverse events and mortality during follow-up compared with men. In the predictor analysis, male sex was associated with a better long-term outcome. Remarkably, our study had a follow-up of more than 6 years.

A recently published meta-analysis with 28,671 MINOCA patients and a mean follow-up of 2 years reached a similar conclusion^23^. In this analysis, women experienced significantly more adverse events than men, although overall mortality rates did not differ^23^. A study by Canton et al. included 2,455 patients with acute myocardial infarction undergoing coronary angiography^24^. Patients were categorized based on myocardial infarction type (MINOCA or with obstructive CAD) and sex^24^. Unadjusted adverse events incidence was higher in females than males, both in MINOCA and with obstructive CAD cohorts, over a median follow-up of 28 months^24^.

Taking these results together with our study, there is evidence that women have a worse long-term outcome after MINOCA than men.

Thus, the question arises as to whether patient characteristics at the time of MINOCA or both acute or long-term treatment might explain the gender difference in adverse event rates, or whether there are other gender-dependent pathomechanisms in MINOCA that have not yet been conclusively clarified.

Both echocardiographic parameters such as left ventricular ejection fraction and laboratory parameters as objective markers of myocardial damage, e.g., troponin and creatine phosphokinase, did not differ between men and women in our cohort. In addition, men were significantly more likely to have electrocardiographic ST-segment elevation. Hence, one would expect that the men perhaps initially had greater myocardial damage than women. These objective parameters cannot therefore be reconciled with the worse outcome in women.

Our study, as well as other studies in the past, have demonstrated that female MINOCA patients are older than males, possibly due to a decreasing protective oestrogen effect^14,23,25^. Another common finding among MINOCA studies and our study is that advanced age is associated with worse outcomes and higher mortality rates^18,20^. Older age is usually associated with higher comorbidity and mortality rates, even in the absence of MINOCA, which might be considered as an explanation for the higher adverse event rate and the increased mortality in female MINOCA patients in the present study cohort.

Another important contributing factor to the increased mortality in females in our study cohort may have been the rate of malignancies. Female MINOCA patients presented with a significantly higher rate of malignant disease than males. In addition to the significantly older age of women, this could be another baseline characteristic responsible for the increased mortality, predominantly non-cardiovascular. However, malignancy did not represent a predictor of out-of-hospital adverse events in the overall study cohort.

Previous studies have demonstrated that female sex and cancer are predictors of MINOCA^26^. Cancer may predispose to or even induce MINOCA, e.g., oncologic therapy with cardiotoxic medication or radiotherapy^27^. A recent meta-analysis, based on data from 9 studies encompassing 26,636 patients, exhibited that 2.5% of MINOCA patients had a malignancy diagnosis at presentation^28^. Interestingly, malignancy was more prevalent in MINOCA compared to myocardial infarction with CAD^28^. Over a median follow-up of 39 months, 7.8% of MINOCA patients died, and meta-regression analysis highlighted associations between long-term mortality and malignancy at presentation, suggesting that malignancy in MINOCA is not insignificant and is linked to an unfavourable long-term prognosis^28^. In another study involving 1,011 consecutive myocardial infarction patients undergoing coronary angiography, 72 were diagnosed with MINOCA^29^. Among MINOCA patients, 29.2% had active cancer compared to 12.0% in those with myocardial infarction and obstructive CAD^29^. Standardized mortality rates were significantly higher in cancer MINOCA (26.7%/year), and cancer myocardial infarction with obstructive CAD (25.0%/year) compared to their non-cancer counterparts, while active cancer was independently associated with higher long-term mortality^29^.

Interestingly, cancer associated MINOCA patients exhibited higher rates of Takotsubo syndrome compared to non-cancer MINOCA patients^29^. Further studies have also indicated that female gender in combination with cancer is associated with the occurrence of Takotsubo syndrome and that Takotsubo patients with malignant disease exhibit significantly increased long-term adverse event and mortality rates^30–34^. Furthermore, there are two meta-analyses exploring the association between Takotsubo syndrome and malignancy. The first meta-analysis aimed to evaluate the clinical outcome of Takotsubo syndrome patients with cancer^35^. It included four studies with a total of 123,563 patients, revealing a prevalence of 6.7% of current or previous malignancy in Takotsubo syndrome patients^35^. Compared to control patients, those with cancer had a significantly increased risk of adverse events both in-hospital and at follow-up^35^. The second meta-analysis focused on the predictive value of malignancy for the prognosis of Takotsubo syndrome patients^36^. It included ten studies and found that malignancy was associated with higher mortality in Takotsubo syndrome patients^36^. Subgroup analysis indicated that this predictive value held for both in-hospital death and follow-up death^36^. The meta-analyses suggested that malignancy plays a significant role in predicting the risk of adverse events and mortality of Takotsubo syndrome patients in both the short and long term^35,36^.

Moreover, the gender-related differences and cardiovascular co-morbidities could potentially have an influence on the long-term outcome. In our predictor analysis, age ≥ 70 years, arterial hypertension, diabetes mellitus, supraventricular tachycardia, pulmonary disease, neurological disease, and kidney disease were identified as predictors of out-of-hospital adverse events, whereas male sex was associated with a better long-term outcome. These results indicate that sex does have an influence on long-term outcome after MINOCA, but cardiovascular and non-cardiovascular co-morbidities do also play a major role. Perhaps gender may not be the decisive factor than the composition of the female and male cohorts with their respective distributions of co-morbidities.

Interestingly, men were not more likely to receive antiplatelet medication than women, although they were significantly more likely to present electrocardiographically with ST-segment elevation, which might have led treating physicians to initiate therapy with aspirin and/or a P2Y12 inhibitor more frequently. In the present study, we could not confirm an undertreatment of one sex compared with the other. However, this does not necessarily indicate whether there might have been a general undertreatment or whether certain subgroups of patients, e.g., older women with cancer, might have benefited more from a different or intensified medication strategy.

Studies on therapy after MINOCA are scarce. Consistent with our study, Gao et al. did not report a difference in treatment between men and women in their MINOCA study of 1179 patients, however, the adverse event rates during follow-up were also not different between the sexes^14^. Another study investigated sex differences in the presentation, treatment, and outcomes of myocardial infarction in individuals under the age of 50 ^37^. Among 2097 patients, including 404 females, women were more likely to have MINOCA^37^. Treatment disparities were observed, with women less likely to be prescribed aspirin, beta-blockers, angiotensin-converting enzyme inhibitors/angiotensin-receptor blockers, and statins^37^. Although in-hospital mortality did not significantly differ, women who survived to discharge had a higher all-cause mortality rate over a median follow-up of 11.2 years, with no significant difference in cardiovascular mortality^37^.

Similar to other gender-differentiating studies on MINOCA, this study suggests that a better understanding of the underlying mechanisms contributing to these gender differences is needed^37^. Hormonal factors, hypercoagulability, smaller blood vessels, as well as under-diagnosis and undertreatment may contribute to the complexity of MINOCA and the increased adverse event rates in women^23,38^. Further large prospective studies are required to better understand the pathophysiological mechanisms of gender difference in MINOCA and to provide more individualized patient´s treatment to reduce subsequent adverse events and mortality rates.

## Limitations

The retrospective nature of the present study is an important limiting factor. In addition, there is heterogeneity within the study cohort, which may affect the generalizability of our results. The inclusion of patients with a working diagnosis of MINOCA adds additional complexity as a potential confounder, as differences in final diagnoses and therapies within this cohort may have influenced the observed outcomes. It is conceivable that patients may have received a definitive diagnosis during hospitalization, such as myocarditis or Tako-Tsubo syndrome, which was not considered in this analysis.

This study involved a monocenter approach, which could have implications for medications prescribed at discharge. The single-center focus raises questions regarding the applicability of our findings to broader MINOCA populations. In addition, the cohort is relatively small, complicating the detection of statistically significant differences and potentially limiting the robustness of some comparisons.

In addition, the lack of a control group with obstructive CAD is a notable limitation.

However, it should be emphasized that the follow-up period is very long compared to other studies and that several adverse events occurred, so that complications and events could also be systematically recorded in detail over the long-term course and not just over a short period during or after hospitalization.

## Conclusion

In this study of 373 troponin-positive patients with non-obstructive CAD, we examined the prognostic impact of gender on in- and out-of-hospital adverse events and long-term outcomes, including mortality. In summary, while in-hospital complications did not show significant gender disparities, females exhibited a higher incidence of adverse events including mortality in the long-term follow-up compared to males. In addition, our results highlight that both cardiovascular and non-cardiovascular comorbidities significantly impair the outcome after MINOCA. In the future, large prospective studies are urgently required to improve our understanding of the pathophysiological mechanisms of gender differences in MINOCA and to enable more individualized treatment of patients to prevent them from subsequent adverse events and to reduce overall mortality.

## Data Availability

Data available on request due to privacy/ethical restrictions. The data that support the findings of this study are available on request from the corresponding author. The data are not publicly available due to privacy or ethical restrictions.
